# Trends in Crimean–Congo Hemorrhagic Fever in Iraq, 2023–2025: A Descriptive Ecological Study of National Surveillance Data and Public Health Response Activities

**DOI:** 10.1002/hsr2.73045

**Published:** 2026-08-26

**Authors:** Sirwan Sleman, Zaniar A. Abass, Masood B. Ameen, Omed I. Abid, Barham J. Abdullah

**Affiliations:** ^1^ College of Veterinary Medicine University of Sulaimani Sulaymaniyah Iraq; ^2^ Masi Altuni Company Sulaymaniyah Iraq; ^3^ Sulaimani Private Veterinary Hospital Sulaymaniyah Iraq

## Abstract

**Background:**

Crimean–Congo hemorrhagic fever (CCHF) remains a significant zoonotic and public health threat in endemic regions, including Iraq, which has experienced repeated outbreaks over the past decade. Recent surveillance data suggest evolving transmission dynamics influenced by climatic variability, ecological conditions, and the intensity of public health interventions.

**Objective:**

This study aimed to describe the epidemiological trends of laboratory‐confirmed CCHF cases in Iraq from 2023 to 2025, characterize the distribution of cases across time and provinces, and assess the potential influence of seasonal factors and combined risk communication and community engagement (RCCE) interventions.

**Methods:**

A descriptive ecological study was conducted using aggregated laboratory‐confirmed Crimean–Congo hemorrhagic fever (CCHF) surveillance data collected by the Iraqi Ministry of Health and supplemented with reports from the World Health Organization (WHO) and collaborating public health partners. Temporal and geographic distributions of confirmed cases reported during 2023–2025 were summarized descriptively. Public health response activities, including risk communication and community engagement (RCCE), vector control, laboratory strengthening, and clinical preparedness, were reviewed to provide epidemiological context. No analytical methods were performed to evaluate intervention effectiveness or infer causal relationships.

**Results:**

Although Iraq remains endemic for CCHF, a marked reduction in confirmed cases was observed in 2024 and 2025 compared with 2023. The decline was particularly evident during early transmission periods and coincided with delayed seasonal onset, increased rainfall, and the early implementation of coordinated multisectoral prevention campaigns initiated in 2024/2025. Enhanced collaboration between human and veterinary health sectors, expanded laboratory diagnostic capacity, and intensified public awareness campaigns were temporally associated with this reduction.

**Conclusion:**

The observed decline in CCHF burden during 2024/2025 compared with 2023 coincided with intensified multisectoral interventions, particularly strengthened RCCE activities. These findings underscore the critical role of early, coordinated, and climate‐sensitive public health responses, as well as sustained RCCE strategies, in mitigating CCHF transmission in the absence of a licensed human vaccine. Continued cross‐sectoral collaboration among human, animal, and environmental health systems is essential to prevent future outbreaks in Iraq.

## 1 Introduction

Crimean–Congo hemorrhagic fever (CCHF) is a severe and potentially fatal tick‐borne viral disease caused by the Crimean–Congo hemorrhagic fever virus (CCHFV), a member of the *Bunyaviridae* family [1]. Transmission occurs primarily through the bite of infected *Hyalomma* ticks or via direct contact with the blood or tissues of infected animals or humans, placing livestock handlers, abattoir workers, and healthcare personnel at particularly high occupational risk [1, 2, 3].

CCHF is endemic across a broad geographic range encompassing much of Africa [4], the Balkans, the Middle East, and parts of Asia. In Iraq, recurrent outbreaks have been reported in recent years, driven by a complex interaction of ecological, climatic, occupational, and health system factors [5, 6]. Case incidence typically shows marked seasonality, with increases during warmer months that coincide with intensified tick activity and greater human exposure through agricultural and animal husbandry practices.

In 2023, Iraq experienced a substantial surge in reported CCHF cases, prompting intensified national and international response efforts. These measures included enhanced surveillance, laboratory capacity strengthening, clinical preparedness, and the implementation of extensive risk communication and community engagement (RCCE) activities. The response was coordinated by the Ministry of Health (MoH) in collaboration with the Ministry of Agriculture (MoA), the World Health Organization (WHO), the Food and Agriculture Organization (FAO), and other partners. Collectively, these actions aligned with the One Health approach, recognizing the interconnectedness of human, animal, and environmental health in the prevention and control of zoonotic diseases [7].

Preliminary surveillance data from the 2024 and early 2025 outbreak seasons indicate a notable decline in reported CCHF cases compared with the 2023 peak. This reduction is likely multifactorial, reflecting the early implementation of preventive veterinary and public health campaigns beginning in April 2024–2025, alongside environmental influences such as delayed summer onset and increased rainfall, which may have affected tick ecology. Additionally, intensified RCCE interventions—including targeted health messaging, engagement of community and religious leaders, and expanded information, education, and communication (IEC) activities—may have contributed to reduced exposure risk and improved early health‐seeking behavior.

Despite these encouraging trends, CCHF remains a persistent public health threat in Iraq, given ongoing challenges related to climate variability, livestock movement, and constraints within the healthcare system. Systematic documentation and analysis of evolving epidemiological patterns and response strategies are therefore essential to inform future preparedness and control efforts. Accordingly, the objective of this manuscript is to describe CCHF epidemiological trends in Iraq during the 2023 and 2024/2025 outbreak seasons and to contextualize these trends within the framework of multisectoral response measures.

## 2 Methods

### 2.1 Study Design and Setting

This study employed a descriptive ecological study design using secondary analysis of routinely collected, aggregated national surveillance data on laboratory‐confirmed Crimean–Congo hemorrhagic fever (CCHF) cases in Iraq. The surveillance data were obtained from the Iraqi Ministry of Health (MoH), together with publicly available reports from the World Health Organization (WHO) and collaborating public health partners.

The study aimed to describe temporal and geographical trends in confirmed CCHF cases during 2023–2025 and to summarize major public health response activities implemented during the same period. Because only aggregated surveillance data were analyzed, the study was descriptive in nature and was not designed to evaluate intervention effectiveness or establish causal relationships between public health interventions and observed epidemiological trends.

### 2.2 Data Sources

Laboratory‐confirmed CCHF surveillance data were obtained primarily from the Iraqi Ministry of Health (MoH) surveillance system and World Health Organization (WHO) situation reports. These official sources constituted the primary epidemiological dataset used for descriptive analyses. Published peer‐reviewed literature was consulted to provide scientific background and contextual interpretation. Publicly available media reports were used only when official surveillance updates were temporarily unavailable and solely to supplement publicly released information. Whenever possible, information obtained from media reports was verified against official reports issued by the Iraqi Ministry of Health or the WHO before inclusion.

### 2.3 Case Definition

The confirmed CCHF cases were determined in line with WHO and national MoH guidelines, which included the following: (1) Suspected clinical presentation consistent with CCHF (acute febrile illness with hemorrhagic manifestations or epidemiological risk factors); and (2) Laboratory confirmation by reverse transcription polymerase chain reaction (RT‐PCR) and/or CCHFV‐specific serology performed at designated national or reference laboratories (WHO). Quantitative analysis involved only laboratory‐confirmed cases.

### 2.4 Data Analysis

Descriptive epidemiological methods were used to summarize monthly, annual, and provincial distributions of laboratory‐confirmed CCHF cases. Frequencies, proportions, percentage changes, and temporal trends were presented using tables and graphical visualizations. Public health response activities were summarized narratively to provide a contextual interpretation of surveillance findings. Because only aggregated surveillance data were analyzed, no inferential statistical analyses, regression models, or causal analyses were performed.

### 2.5 AI Use Statement

No artificial intelligence (AI) tools were used for data collection, data analysis, statistical analysis, or interpretation of the findings.

### 2.6 Review of Public Health and RCCE Interventions

A narrative description of key response activities initiated during the study period was documented. These included:

1. MoH and MoA are working together in inter‐agency coordination in the multisectoral area.
2. Vector control and veterinary health promotion campaigns were initiated in early April 2024/2025.
3. Planned RCCE activities in coordination with WHO, including personalized health messages, social media promotions, community influencer involvement, and working with religious endowments.
4. Activities for capacity building, including tick sequencing and CCHFV laboratory workshops, and capacity development activities.
5. Clinical readiness measures, including the stock and acquisition of ribavirin in syrup, capsule, and injectable formulations.

These activities were measured in the context of historical and longitudinal measurements of their timing with the current epidemiological trends (Table S1).

### 2.7 Statistical Analysis

Descriptive statistical analyses were performed using Microsoft Excel 365 (Microsoft Corp., Redmond, WA, USA) and GraphPad Prism version 10.0 (GraphPad Software, San Diego, CA, USA). Monthly and provincial distributions of laboratory‐confirmed CCHF cases were summarized using frequencies, proportions, percentages, and incidence rates (cases per 100,000 population), where population data were available. Percentage changes between surveillance periods were calculated descriptively. Because this study analyzed aggregated surveillance data using a descriptive ecological design, no inferential statistical tests, regression analyses, time‐series analyses, or hypothesis testing were performed; therefore, no P values or confidence intervals are reported.

## 3 Results

### 3.1 Temporal Trends of Confirmed CCHF Cases (2023–2025)

**Figure 1:**
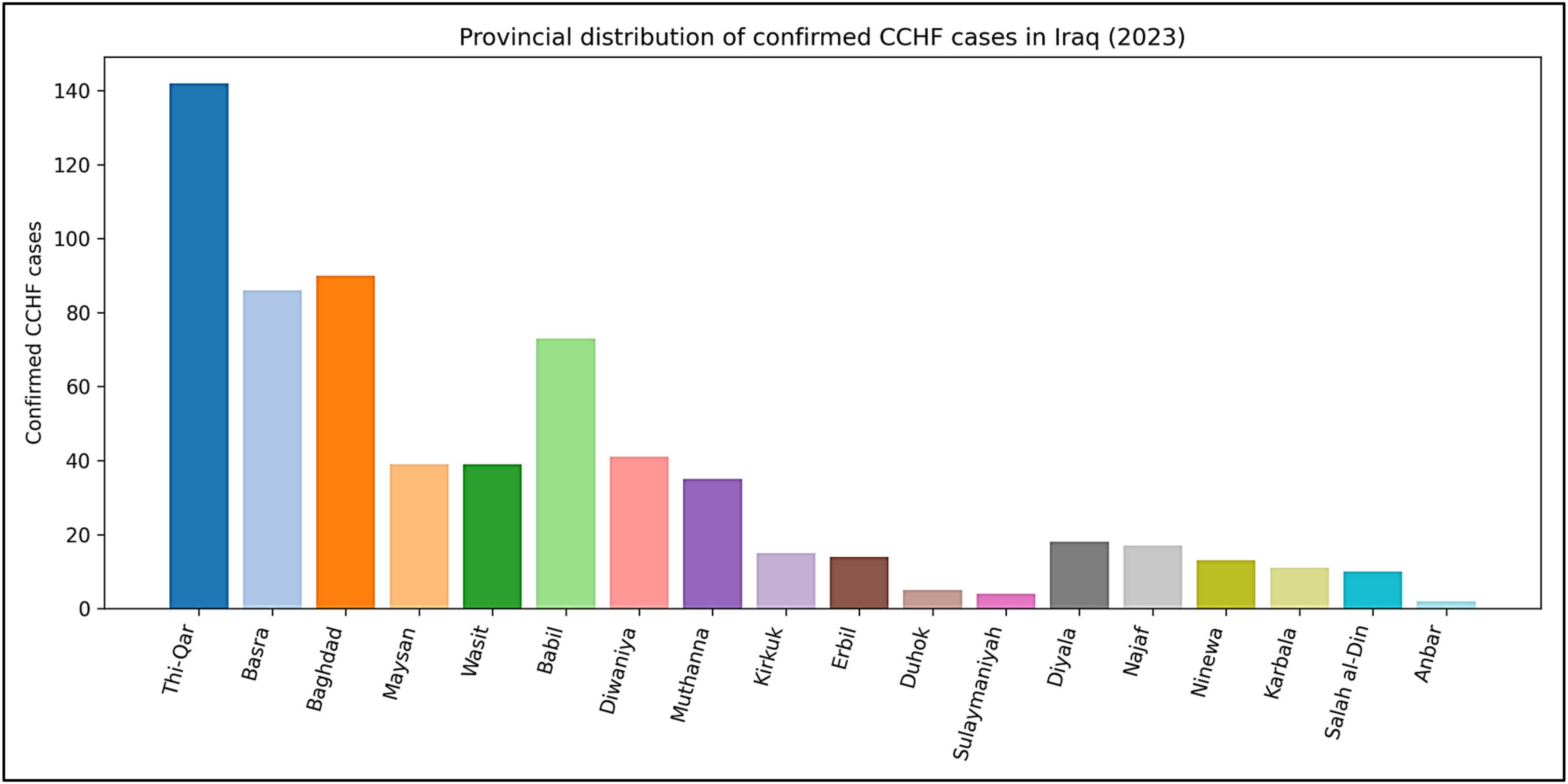
Temporal trends of confirmed CCHF cases by province in 2023. The bar chart depicts the provincial distribution of confirmed CCHF cases in Iraq during 2023, the year with the highest overall case count. Dhi Qar again recorded the greatest burden, followed by notable increases in Basra and Baghdad, while northern provinces reported comparatively low numbers. Each bar represents a governorate and is displayed in a distinct color to improve visual discrimination across provinces.

**Figure 2:**
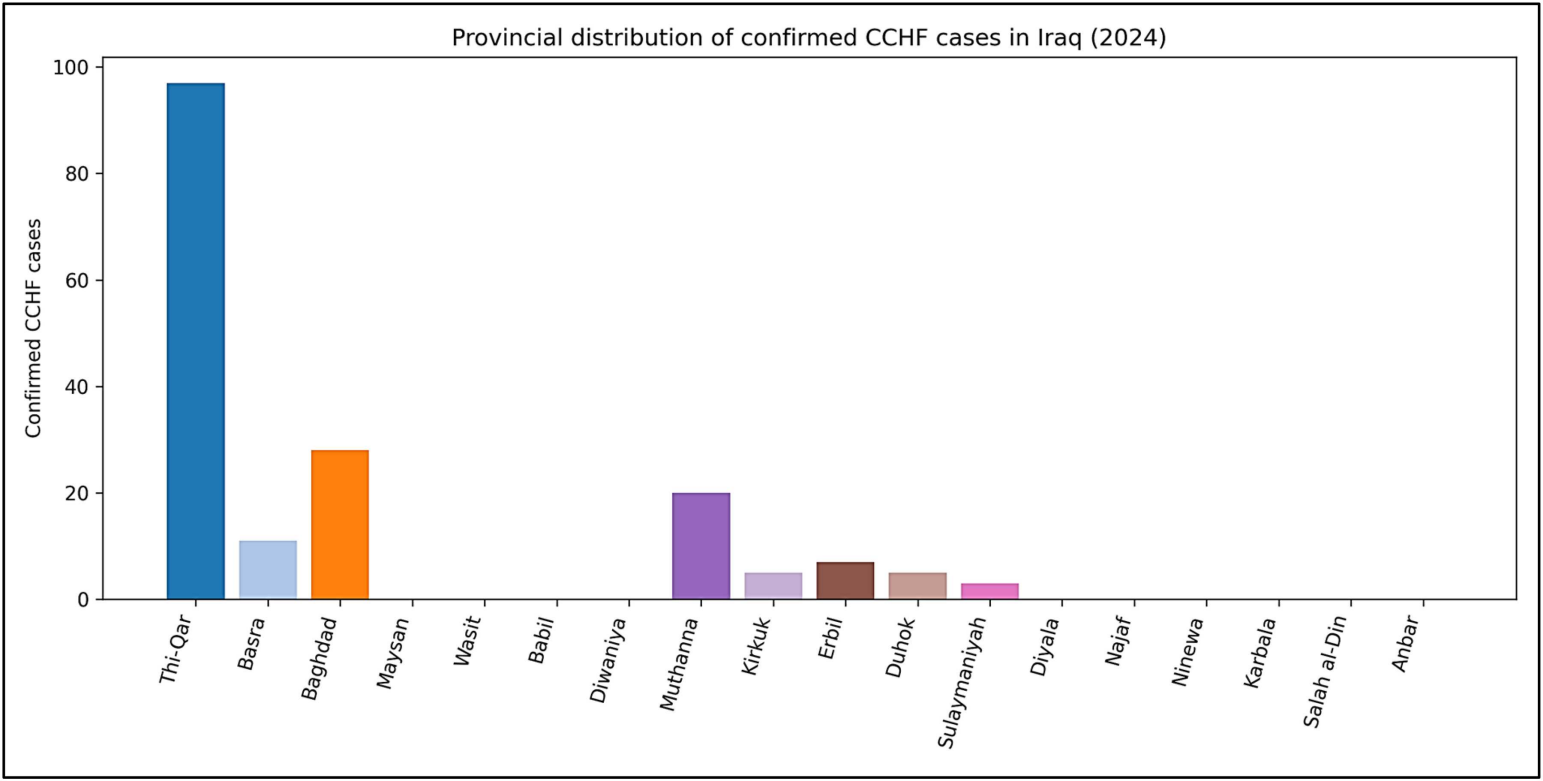
Temporal trends of confirmed CCHF cases by province in 2024. The bar graph presents a significant decline in confirmed CCHF cases for the provinces of Iraq in 2024, with only some places (Dhi Qar, Muthanna) showing a notable CCHF count. In contrast to the much higher rates reported in 2023.

**Figure 3:**
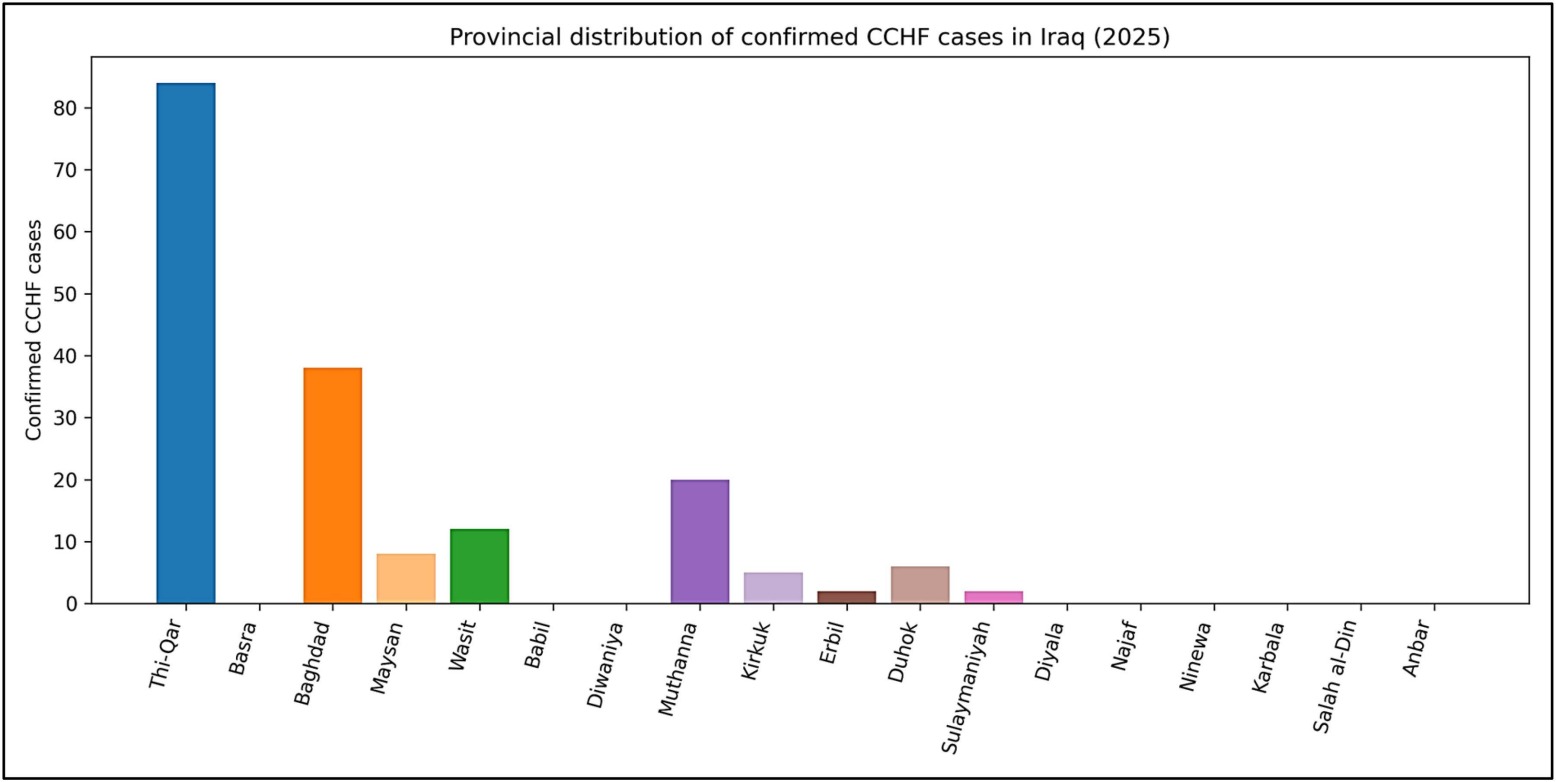
Temporal trends of confirmed CCHF cases by province in 2025. The bar chart indicates that confirmed CCHF cases in 2025 were almost similar to a pronounced drop in 2024, and that Dhi Qar accounted for the overwhelming majority of infections. Nevertheless, the collective incidence rate is still less than the peaks of 2023.

**Figure 4:**
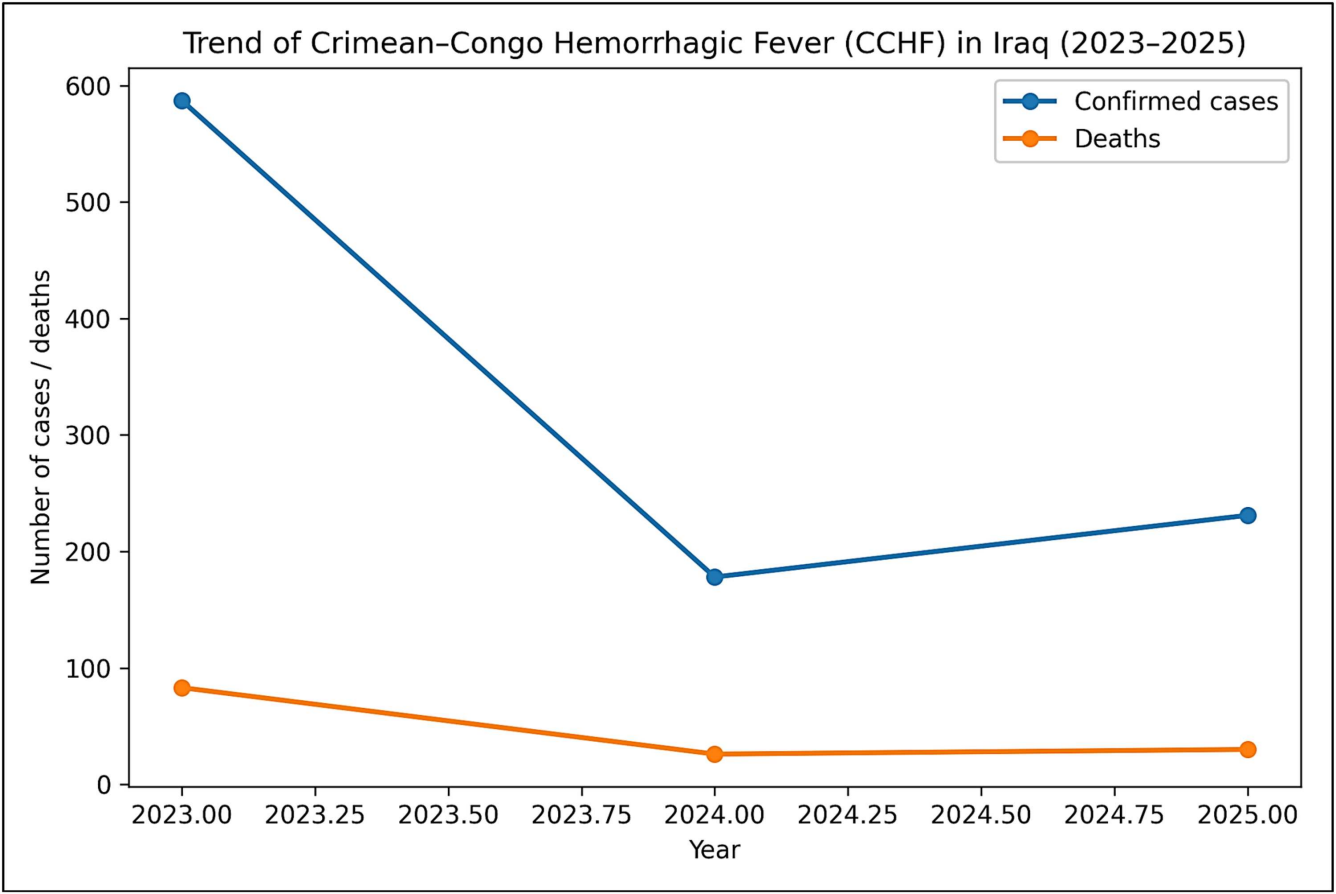
Total trend of Crimean–Congo hemorrhagic fever (CCHF) in Iraq from 2023 to 2025. This figure illustrates the three‐year trends of CCHF in Iraq, showing a sharp rise in both confirmed cases and deaths in 2023, followed by a substantial decline in 2024 and a partial rebound in 2025, with overall levels remaining below the 2023 peak.

Analysis of national surveillance data and International participants revealed significant changes in the number of confirmed CCHF cases reported in the 3 years 2023, 2024, and 2025. In 2023, Iraq experienced a relatively high CCHF burden, significantly between spring and early summer months, a pattern that is in line with established seasonal effects of tick activity and increased human–animal contact. Comparatively, confirmed cases by the epidemiological data in 2024/2025 show a significant decline in the total number of confirmed cases compared to the corresponding same period of reporting in 2023 (Table 1) (Figures 1, 2, 3, 4 **)**. Particularly, the drop observed during the early months of intensive activity of RCCE in 2024/2025 indicates a potential role of this community in the suppression of a classical seasonal increase in cases in the previous years.

**Table 1:** Provincial distribution of confirmed CCHF trends in Iraq.

Governorate (province)	2023 [8]	2024 [9]	2025 [10]	Total cases recorded*
Thi‐Qar (Dhi‐Qar)	142	97	≥ 84	≥ 323
Basra	86	11	NA	97
Baghdad	90	28	38	156
Maysan (Missan)	39	NA	≥ 8	≥ 47
Wasit	39	NA	≥ 12	≥ 51
Babil (Babylon)	73	NA	NA	73
Diwaniya (Al‐Qadisiyah)	41	NA	NA	41
Muthanna	35	≥ 20	≥ 20	≥ 75
Kirkuk	15	5	5	25
Erbil	14	7	2	23
Duhok	5	5	6	16
Sulaymaniyah	4	3	2	9
Diyala	18	NA	NA	18
Najaf	17	NA	NA	17
Ninewa (Nineveh)	13	NA	NA	13
Kerbala (Karbala)	11	NA	NA	11
Salah al‐Din	10	NA	NA	10
Anbar	2	NA	NA	2
Total (national)	587	≥ 178	≥ 231	≥ 996

*Note:* This table summarizes the annual distribution of officially confirmed CCHF cases across Iraqi provinces from 2023 to 2025, totaling 996 cases. Dhi Qar consistently recorded the highest number of cases each year, contributing nearly 40% of all infections. Basra, Baghdad, Babil, Muthanna, and Maysan also demonstrated significant case burdens, whilst northern provinces like Duhok, Erbil, Sulaymaniyah, and Kirkuk had comparatively lower numbers. Data for some provinces in 2024–2025 remain not available (NA) or not reported officially, but the trend appears consistent with the continued concentration of CCHF cases in southern and central Iraq. *Totals reflect minimum confirmed cases only. Ranges (≥) indicate progressive. Data were collated from peer‐reviewed surveillance analyses (2023) and Ministry of Health statements, regional CDC reports, and verified media briefings (2024–2025). Where complete annual governorate‐level data were unavailable, minimum estimates or ranges are presented to reflect reporting uncertainty. National totals, therefore, represent conservative minimum counts.

### 3.2 Provincial Distribution of Cases (2022–2025)

**Figure 5:**
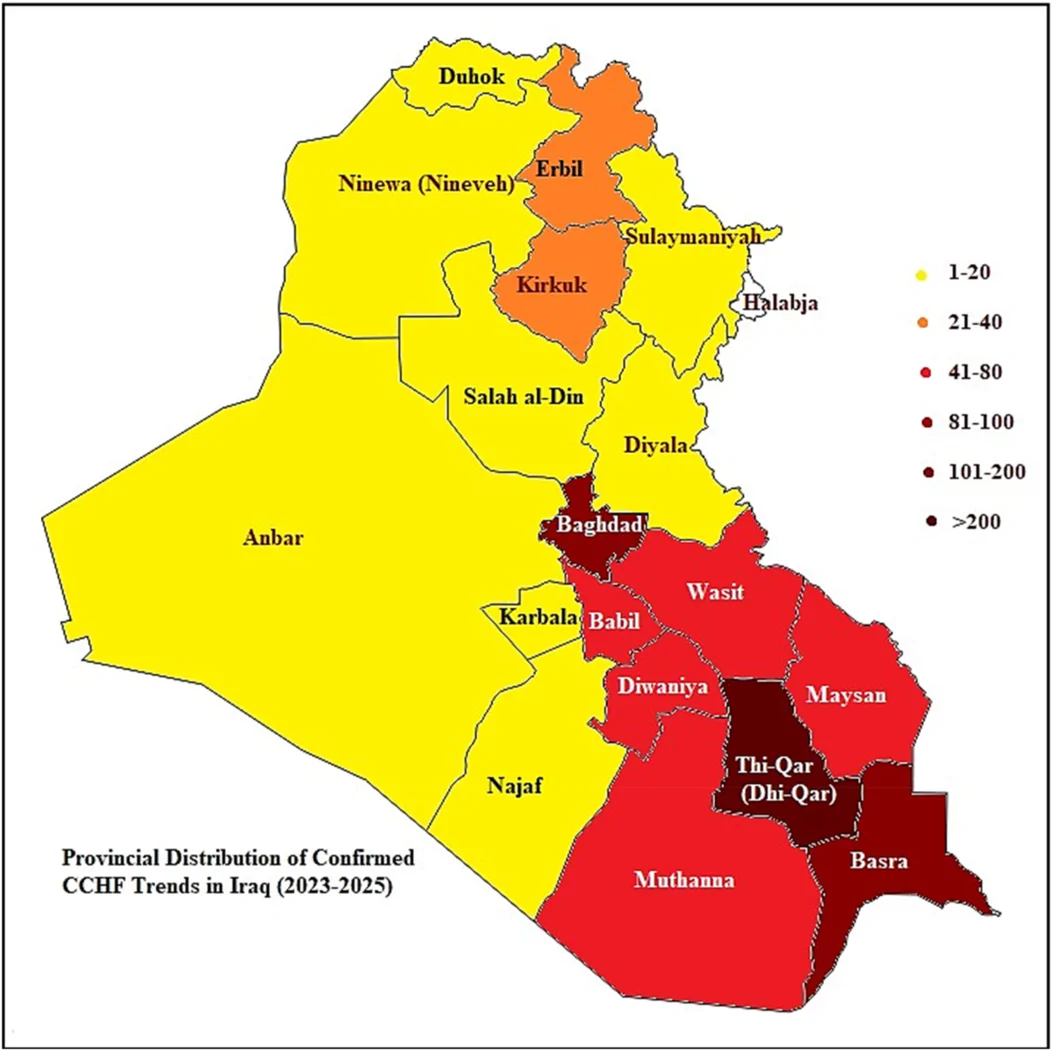
Geographic distribution of total confirmed CCHF cases by province in Iraq, 2023–2025. This map illustrates a geographic overview of total confirmed Crimean–Congo hemorrhagic fever (CCHF) cases across Iraqi provinces during 2023–2025, showing marked spatial heterogeneity. Higher burdens are concentrated in central and southern provinces—particularly Baghdad, Basra, Thi‐Qar, and parts of the Middle Euphrates—while northern and western provinces report comparatively lower case numbers. Overall, the pattern highlights persistent endemic foci in southern Iraq with limited spread into the north over the study period.

**Figure 6:**
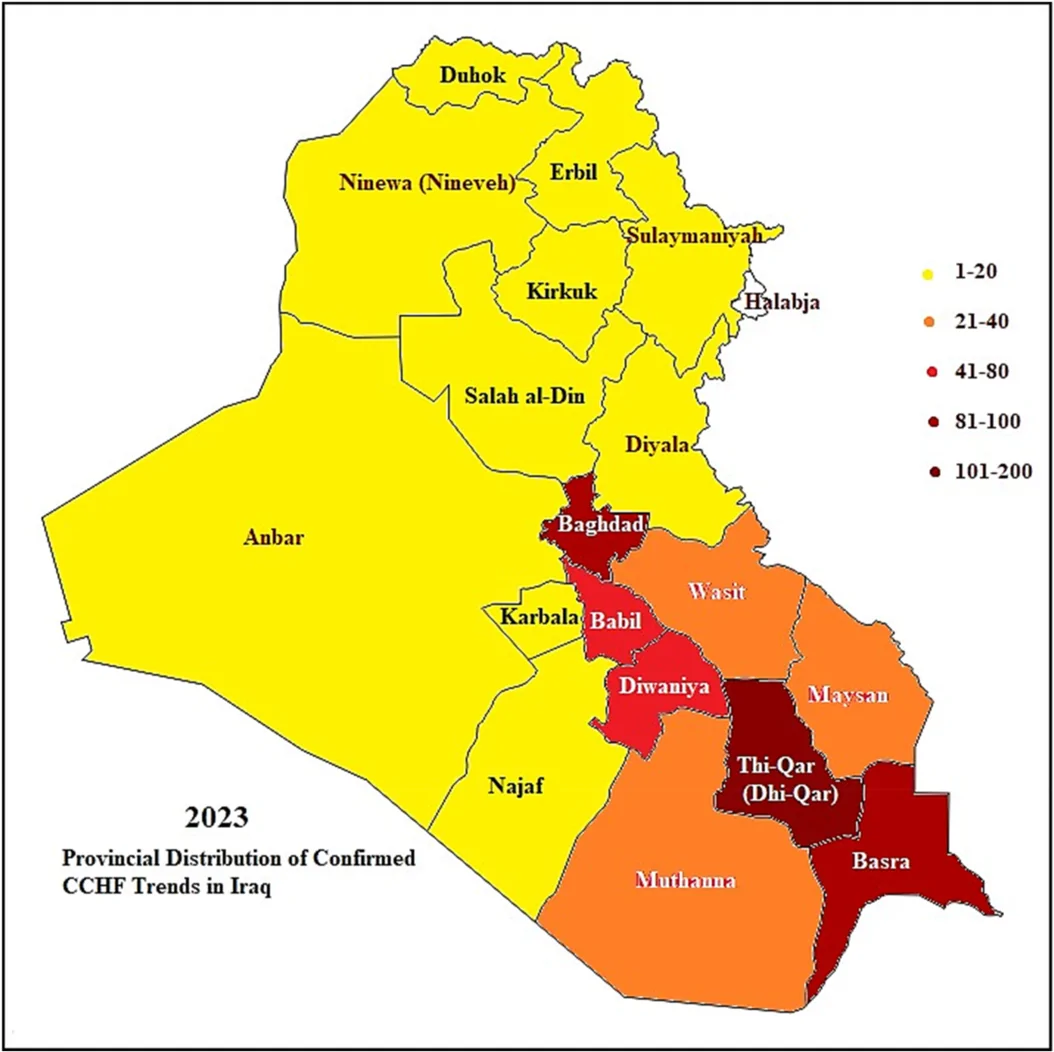
Geographic distribution of total confirmed CCHF cases by province in Iraq, 2023. This figure shows the geographic distribution of total confirmed Crimean–Congo hemorrhagic fever (CCHF) cases across Iraqi provinces during 2023, revealing clear regional variation. The highest case burdens are concentrated in central and southern provinces, particularly Baghdad, Basra, Thi‐Qar, and Diwaniya, whereas northern and western provinces report substantially lower case counts. Overall, the pattern indicates a strong south‐central focus of CCHF transmission in Iraq.

**Figure 7:**
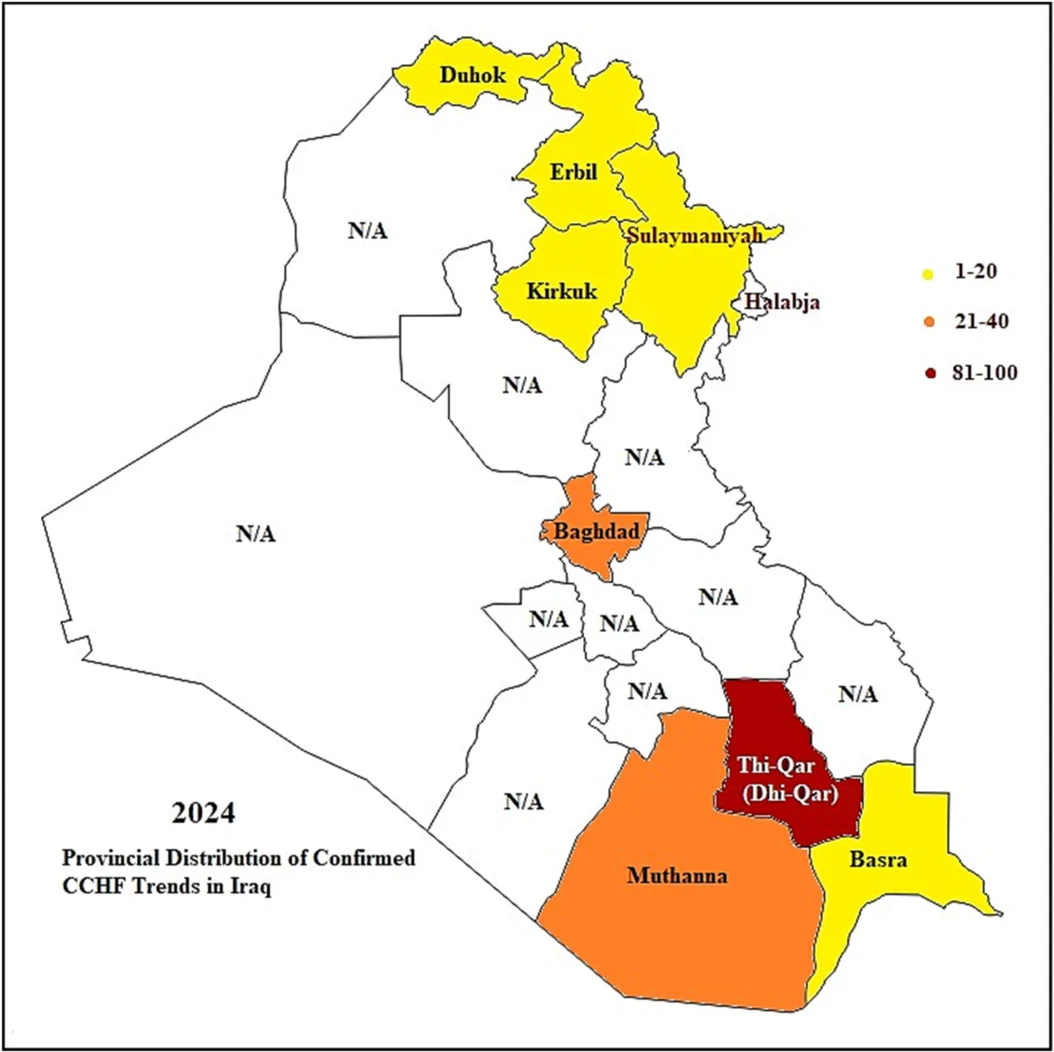
Geographic distribution of total confirmed CCHF cases by province in Iraq, 2023–2025. This figure shows the geographic distribution of confirmed Crimean–Congo hemorrhagic fever (CCHF) cases in Iraq in 2024, demonstrating a marked contraction compared with the widespread transmission observed in 2023. Case reporting in 2024 is largely confined to a few southern and central provinces, with Thi‐Qar remaining a high‐burden hotspot and lower‐level activity in Muthanna, Basra, and Baghdad, while many provinces report no available data or no confirmed cases. Overall, the pattern suggests a substantial decline and spatial concentration of CCHF cases in 2024 relative to 2023.

**Figure 8:**
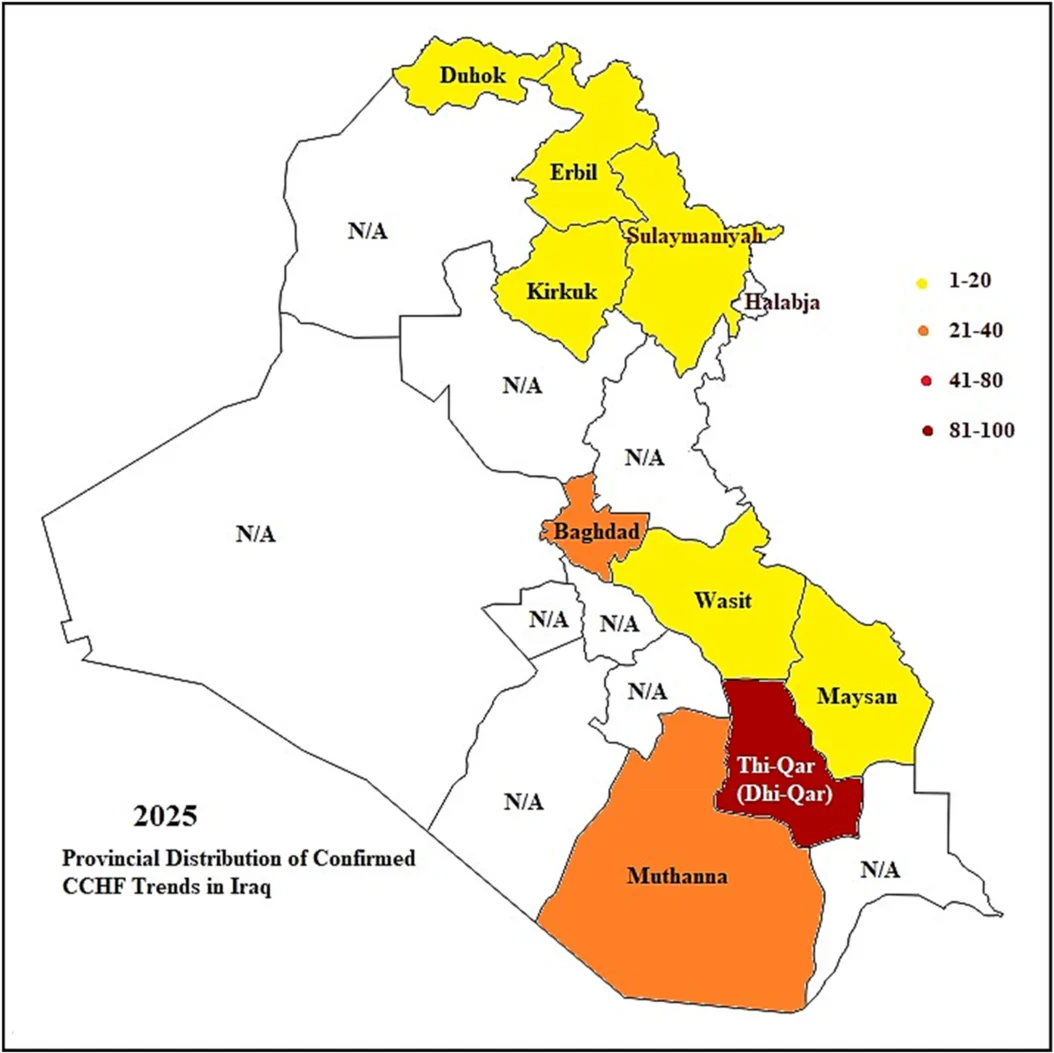
Geographic distribution of total confirmed CCHF cases by province in Iraq, 2025. This figure illustrates the geographic distribution of confirmed Crimean–Congo hemorrhagic fever (CCHF) cases in Iraq in 2025, showing a further reduction compared with 2023, similar to that of 2024 cases, with a modest increase. While 2023 exhibited widespread transmission across central and southern provinces, cases in 2025 are largely limited to southern Iraq, with Thi‐Qar remaining the principal hotspot and lower‐level activity in Muthanna, Maysan, Wasit, and Baghdad. Overall, the pattern indicates a sustained decline and increasing localization of CCHF transmission over the study period.

The range of confirmed CCHF cases between 2023, 2024, and 2025 exhibited cross‐provincial variation in geographic distribution. Provinces that had long suffered continued to report cases, but in 2024/2025, dissemination of cases was at a lower level (Table 2) (Figures 5, 6, 7, 8 **)**.

**Table 2:** Prevalence of confirmed CCHF cases.

	Total confirmed CCHF cases and references	Deaths (approx.)
2023	587 [9]	83
2024	178 [10]	26
2025	231 [8]	42

*Note:* This table summarizes the prevalence of confirmed CCHF cases in Iraq from 2023 to 2025, based on available surveillance data, published studies, media/health reports and available WHO/CDC reports.

Livestock‐rich and animal‐trade regions remained areas of concern but were not significant epidemiological concerns. With a similar picture in livestock‐and‐animal trade‐rich/highly affected provinces, the number of confirmed tyr6578b cases from these provinces was slightly below the expected based on previous seasonal trends. This result indicates a possible role of early preventive measures and environmental elements in reducing the risk of transmission.

### 3.3 Public Health and Veterinary Interventions

The following interventions were performed before and early in the year 2024 to 2025. Since 1 April 2024, the Ministry of Agriculture (MoA)‐led veterinary and agricultural campaign activities aimed at controlling ticks, livestock, and also increasing pet husbandry knowledge in animals and handlers. At the same time, the Ministry of Health (MoH), with the support of the World Health Organization (WHO) and its partners, encouraged additional RCCE activities.

Key RCCE interventions were organizing meetings with health promotion agencies, the development and communication of culturally relevant CCHF messages, dissemination and use of content messages, social media packages, and engagement with community and religious influencers. WHO and FAO also supported the production of information, education and communication (IEC) materials for distribution in the field. Altogether, RCCE interventions and activities played a potential role in reducing CCHF cases in the region.

### 3.4 Laboratory and Clinical Preparedness

Measures to build capacity in diagnostics and response were undertaken. A tick sequencing and CCHFV workshop was held with both the MoH and the MoA, and was performed at the Veterinary Central Laboratory and Central Public Health Laboratory. In clinical terms, ribavirin, the main antiviral therapeutic for CCHF cases, was identified as accessible across the MoH drug supply chain and procured further in collaboration with partner organizations for stocking.

### 3.5 Seasonal and Environmental Context

The decrease in CCHF cases in 2024/2025 was also consistent with abnormal seasonal conditions such as late accruals of ambient temperature and heavy precipitation. These contextual factors may reflect dynamics in tick populations, interactions between host and vector and patterns of human exposure. This suggests that the climate in 2024/2025 seemed to be less favorable for the rapid amplification of tick‐borne transmission compared to the peak‐case year, 2023.

## 4 Discussion

This descriptive ecological study summarizes recent national surveillance data on laboratory‐confirmed Crimean–Congo hemorrhagic fever (CCHF) cases in Iraq and describes temporal changes observed during the 2023–2025 surveillance periods. As an observational analysis of aggregated surveillance data, the study was designed to characterize epidemiological patterns and associated public health response activities rather than evaluate the effectiveness of specific interventions or establish causal relationships.

Seasonality has been identified as a well‐established contributor to the transmission of CCHF, and peaks were achieved by warmer months when the activity of *Hyalomma* ticks and human–livestock interactions were increased [1, 2, 3]. The delayed seasonal increase in cases of infection in 2024/2025 corresponded with the later onset of summer temperatures and heavy rains and might have disrupted tick life cycles and reduced effective contact between the vectors, animal hosts, and humans. Previous studies from endemic regions have reported associations between climatic variability, including rainfall and temperature patterns, and seasonal CCHF transmission dynamics. However, because meteorological variables were not quantitatively analyzed in the present study, these observations should be interpreted cautiously and cannot be considered evidence of a causal relationship [11, 12].

The observed decline in reported CCHF cases temporally coincided with strengthened multisectoral response activities, including risk communication and community engagement (RCCE) initiatives, veterinary tick‐control campaigns, enhanced laboratory preparedness, and improved coordination between the Ministry of Health (MoH) and the Ministry of Agriculture (MoA). These activities are consistent with the One Health approach, which emphasizes coordinated action across the human, animal, and environmental health sectors for the prevention and control of zoonotic diseases [7]. However, given the descriptive ecological design of the present study, it is not possible to attribute the observed reduction in CCHF cases directly to these interventions. Rather, the findings demonstrate a temporal association between the implementation of public health response activities and the observed epidemiological trends.

Alternative explanations should also be considered. Changes in surveillance sensitivity, reporting practices, healthcare‐seeking behavior, laboratory diagnostic capacity, underreporting of mild or asymptomatic infections, and environmental variability may all have influenced the observed decline in reported cases [1, 5, 6]. Likewise, previous studies from endemic regions have shown that climatic variability, vector ecology, and livestock movement substantially influence CCHF transmission dynamics [11, 12]. Therefore, the observed reduction in CCHF cases is likely multifactorial, and the present findings should be interpreted as descriptive observations rather than evidence of intervention effectiveness.

Similar temporal patterns have been reported in neighboring endemic countries, including Iran, Turkey, and Saudi Arabia, where fluctuations in CCHF incidence have been associated with complex interactions among vector ecology, livestock movement, climatic variability, surveillance capacity, and public health preparedness. These regional observations support the importance of integrated One Health surveillance while emphasizing that multiple epidemiological determinants, rather than any single intervention, likely contribute to changes in disease incidence [5, 6, 7, 11, 12]. Future analytical studies incorporating epidemiological, climatic, and intervention‐related data are warranted to better quantify the relative contribution of these factors.

Enhanced laboratory and clinical readiness further strengthened the control capacity of outbreaks. Tick sequencing and CCHFV diagnostics training increased intersectoral technical capacity, while ribavirin availability confirmation and procurement preparedness, and readiness to manage the case further strengthened the intersectoral technical resources. The clinical efficacy of ribavirin is debated but remains the recommended option in many endemic settings as part of comprehensive CCHF management protocols [1].

Despite these observations, several limitations should be acknowledged. First, the study relied on aggregated surveillance data, precluding individual‐level analyses of demographic characteristics, risk factors, clinical outcomes, or intervention effectiveness. Second, the descriptive ecological design does not permit causal inference between public health interventions and changes in disease incidence. Third, surveillance data may have been influenced by underreporting, changes in reporting practices, healthcare access, or laboratory diagnostic capacity. Finally, meteorological and ecological variables were not quantitatively analyzed, limiting assessment of their independent contribution to the observed temporal trends.

Overall, this study demonstrates a substantial decline in reported CCHF cases during the corresponding surveillance periods of 2024–2025 compared with 2023. This decline temporally coincided with strengthened multisectoral preparedness activities and environmental variability; however, the observational nature of the study precludes causal attribution. Continued One Health surveillance, strengthened laboratory capacity, climate‐informed preparedness, sustained community engagement, and coordinated public health interventions remain essential for improving CCHF prevention and outbreak preparedness in Iraq.

## 5 Conclusion

This descriptive ecological study provides an updated overview of the epidemiological trends of laboratory‐confirmed Crimean–Congo hemorrhagic fever (CCHF) in Iraq during the 2023–2025 surveillance periods. A substantial reduction in reported CCHF cases was observed during the corresponding surveillance periods of 2024–2025 compared with 2023. This decline temporally coincided with strengthened multisectoral public health preparedness, including risk communication and community engagement (RCCE), veterinary tick‐control activities, enhanced laboratory capacity, and improved intersectoral coordination, together with environmental variability. However, because this study was based on aggregated surveillance data and employed a descriptive ecological design, no causal relationship between these factors and the observed reduction in disease incidence can be established.

The findings highlight the importance of sustaining a coordinated One Health approach that integrates human, animal, and environmental health sectors to strengthen CCHF preparedness and response. Continued investment in climate‐informed surveillance, early vector‐control measures, laboratory diagnostic capacity, standardized reporting systems, and targeted community awareness programmes for high‐risk populations, including farmers, livestock handlers, butchers, and healthcare workers, may enhance early detection, improve outbreak preparedness, and reduce the public health impact of future CCHF outbreaks in Iraq. Future analytical studies incorporating epidemiological, climatic, and intervention‐related data are warranted to better evaluate the determinants of CCHF transmission and the effectiveness of specific public health interventions.

## Public Health Impacts

1. **Significant Reduction in Disease Burden:** According to surveillance records, the confirmed cases of Crimean–Congo hemorrhagic fever (CCHF) in 2024 and 2025 were reduced substantially compared with the previous years of 2023, highlighting the improved multisectoral coordination and effective interventions during the early season.
2. **Strengthened One Health Preparedness:** Enhanced collaboration strategies between human and animal health sectors, including early veterinary tick‐control campaigns, developed diagnostics, and improved clinical readiness, have participated in suppressing transmission and raising outbreak responses.
3. **Improved Public Awareness:** Expanded communication, risk communication, and community engagement (RCCE), such as engagement of social media, community interactions, and religious leaders, potentially reduced the high‐risk exposure and improved the early health‐seeking behavior, thus facilitating continued prevention.

## Recommendations

Based on the observed trends and response activities, several recommendations emerge:

1. **Sustain One Health Coordination:** Further integration between the Ministry of Health and the Ministry of Agriculture in the area of tick control, livestock management, and early outbreak detection is important and needs to continue.
2. **Institutionalize Early RCCE Campaigns:** Risk communication campaigns need to be started every year prior to the expected transmission season and be relevant to local sociocultural contexts.
3. **Strengthen Climate‐Sensitive Surveillance:** Use of meteorological and ecological indicators in routine surveillance can help enhance early warning and preparedness.
4. **Maintain Laboratory and Clinical Readiness:** Continuous support, such as regular training in CCHFV diagnostics and constant availability of ribavirin and supportive care resources, is a must to maintain laboratory and clinical preparedness.
5. **Enhance Data Quality:** Improved case‐based surveillance and digital reporting systems would support detailed analysis of risk factors and outcomes.

## Author Contributions

**Sirwan Sleman:** conceptualization; investigation; funding acquisition; formal analysis; data curation; supervision; resources; validation; methodology; project administration; visualization; software; writing – original draft. **Zaniar A. Abass:** funding acquisition; data curation; formal analysis; writing – review and editing. **Masood B. Ameen:** funding acquisition; data curation; writing – review and editing. **Omed I. Abid:** writing – review and editing; funding acquisition; validation. **Barham J. Abdullah:** funding acquisition; writing – review and editing; software.

## Ethics Statement

This article employed secondary, aggregated surveillance information to support routine public health surveillance data for this study. Thematic analysis included secondary, aggregate data collection from a combined surveillance data collection system and was carried out in the context of secondary public health surveillance during routine public health analysis. No names or identifiers were found. Ethical approval did not necessarily have to be obtained, pursuant to national public health laws and WHO recommendations for routine surveillance use in the event of outbreak data in reporting and analysis.

## Conflicts of Interest

The authors declare no conflicts of interest.

## AI Disclosure

Artificial intelligence‐assisted language editing tools were used only to improve the clarity, grammar, and readability of the manuscript. All scientific content, interpretation of results, and final editorial decisions were independently reviewed and approved by the authors, who take full responsibility for the accuracy and integrity of the manuscript.

## Author Responsibility Statement

All authors have read and approved the final version of the manuscript. **Sirwan Sleman**, as the corresponding author, had full access to all of the data included in this study and takes complete responsibility for the integrity of the data and the accuracy of the data analysis.

## Transparency Statement

Sirwan Sleman affirms that this manuscript is an honest, accurate, and transparent account of the study being reported; that no important aspects of the study have been omitted; and that any discrepancies from the study as originally planned have been fully explained.

## Supporting information


Supporting File

## Data Availability

The data that supports the findings of this study are available in the supporting material of this article.
