# Supplementary material for: Trends in Crimean–Congo Hemorrhagic Fever in Iraq, 2023–2025: A Descriptive Ecological Study of National Surveillance Data and Public Health Response Activities
Source: Health Sci Rep. 2026 Aug 26;9(9):e73045. doi: 10.1002/hsr2.73045 (PMC13519288; doi:10.1002/hsr2.73045)
Supplement: Supplementary file 1 [file HSR2-9-e73045-s001.docx]

**Supplementary Materials**

**Trends in Crimean–Congo Hemorrhagic Fever in Iraq, 2023–2025: A Descriptive Ecological Study of National Surveillance Data and Public Health Response Activities**

**Authors:**

**Sirwan Sleman^1,*^, Zaniar A. Abass^2^, Masood B. Ameen^3^, Omed I. Abid^2^, and, Barham J. Abdullah^3^**

**Affiliations:**

¹College of Veterinary Medicine, University of Sulaimani, Sulaymaniyah, Iraq.

^2^Masi Altuni Company, Sulaymaniyah, Iraq.

^3^Sulaimani Private Veterinary Hospital, Sulaymaniyah, Iraq.

***Corresponding Author:** Assist. Prof. Dr Sirwan Sleman

**Email**: [sirwan.sleman@univsul.edu.iq]

**Phone**: [+9647721525583]

**ORCID** IDs: [Sirwan Sleman (0000-0001-8589-0345) - ORCID](https://orcid.org/0000-0001-8589-0345)

**Running Title:** Trends of CCHF in Iraq

| Period (Year–Month) | Epidemiological Context | Key Public Health Interventions (MoH) | RCCE Interventions | WHO Support & Coordination |
| --- | --- | --- | --- | --- |
| Jan–Feb 2024 | Seasonal preparedness phase; low baseline transmission | National preparedness planning; update of CCHF case definitions; pre-positioning of PPE and ribavirin | Development of risk messages for healthcare workers (HCWs) and high-risk groups | Technical guidance updates, preparedness checklist, and coordination with the MoH surveillance units |
| Mar–Apr 2024 | Early rise in suspected cases (pre-Eid) | Enhanced surveillance in endemic provinces; refresher IPC training for HCWs; abattoir inspections | Targeted awareness for butchers, livestock handlers, and IPC messaging in health facilities | Training materials; rapid risk assessment support; IPC technical assistance |
| May–Jun 2024 | Peak transmission period; Eid Al-Adha | Rapid response teams (RRTs) were activated; contact tracing, infection control audits, and movement control in affected abattoirs. | Mass media campaigns (TV/radio/social media); community leaders engaged; safe slaughter messages | Deployment of surge experts; RCCE toolkits; situation reporting and coordination |
| Jul–Aug 2024 | Sustained transmission in hotspots | Case management support; laboratory capacity strengthening; referral pathway optimization | Myth-busting content; hotline promotion; community dialogues in high-risk districts | Lab diagnostics support; risk communication monitoring; intersectoral coordination |
| Sep–Oct 2024 | Declining cases; post-peak | Surveillance consolidation; after-action reviews (AARs); guideline revisions | Feedback to communities; reinforcement of preventive behaviors | AAR facilitation; documentation of lessons learned |
| Nov–Dec 2024 | Low transmission; inter-season | National review and planning for next season; stock replenishment | Pre-season preparedness messaging; HCW refresher materials | Strategic planning; funding advocacy; preparedness support |
| Jan–Feb 2025 | Pre-season readiness | Updated SOPs; simulation exercises; cross-border information sharing | Pre-emptive risk messaging; stakeholder briefings | Technical updates; cross-border coordination |
| Mar–Jun 2025 | Anticipated seasonal rise | Scaled surveillance; rapid diagnostics; IPC reinforcement; RRT deployment | Expanded RCCE targeting rural/high-risk groups; real-time rumor tracking | RCCE analytics; surge capacity; ongoing technical support |
| Jul–Dec 2025 | Peak then decline | Sustained response transitioning to recovery; health system strengthening | Community feedback loops; sustained behavior change messaging. | Evaluation, reporting, and sustainability planning |

**Table S1. Timeline of Public Health and RCCE Interventions, 2024–2025 (MoH–WHO Aligned).** This table summarizes the timeline of public health and RCCE interventions (2024–2025), aligned with the Iraq Ministry of Health (MoH) and WHO activities. RCCE = Risk Communication and Community Engagement, IPC = Infection Prevention and Control, RRTs = Rapid Response Teams.
